# Furanocembranoid from the Okinawan soft coral *Sinularia* sp.

**DOI:** 10.1007/s13659-022-00330-7

**Published:** 2022-03-02

**Authors:** Misaki Nagasaka, Kazuki Tani, Keisuke Nishikawa, Riri Kinjo, Takahiro Ishii

**Affiliations:** 1 Department of Biosciences and Biotechnology, Faculty of Agriculture, University of the Ryukyus, Senbaru 1, Nishihara, Okinawa 903-0213 Japan; 2 Department of Chemistry, Graduate School of Science, Osaka City University, Osaka, 558-8585 Japan

**Keywords:** Soft coral, *Sinularia* sp., Furanocembranoid, Diterpene

## Abstract

One new furanocembranoid diterpene, 11-hydroxy-Δ^12(13)^-pukalide (**1**), along with six known secondary metabolites, 11-acetoxy-Δ^12(13)^-pukalide (**2**), 13α-acetoxypukalide (**3**), pukalide (**4**), 3α-methoxyfuranocembranoid (**5**), Δ^9(15)^-africanene (**6**), and methyl (5′*E*)-5-(2′,6′-dimethylocta-5′,7′-dienyl)furan-3-carboxylate (**7**) were isolated from the Okinawan soft coral *Sinularia* sp. Their chemical structures were elucidated based on spectroscopic analysis (FTIR, NMR, and HRESIMS), and the relative stereochemistry of **1** was determined by NOESY experiments and acetylation, which yielded derivative **2**. In addition, compounds **1** and **7** exhibited toxicity in the brine shrimp lethality test.

**Graphical Abstract:**

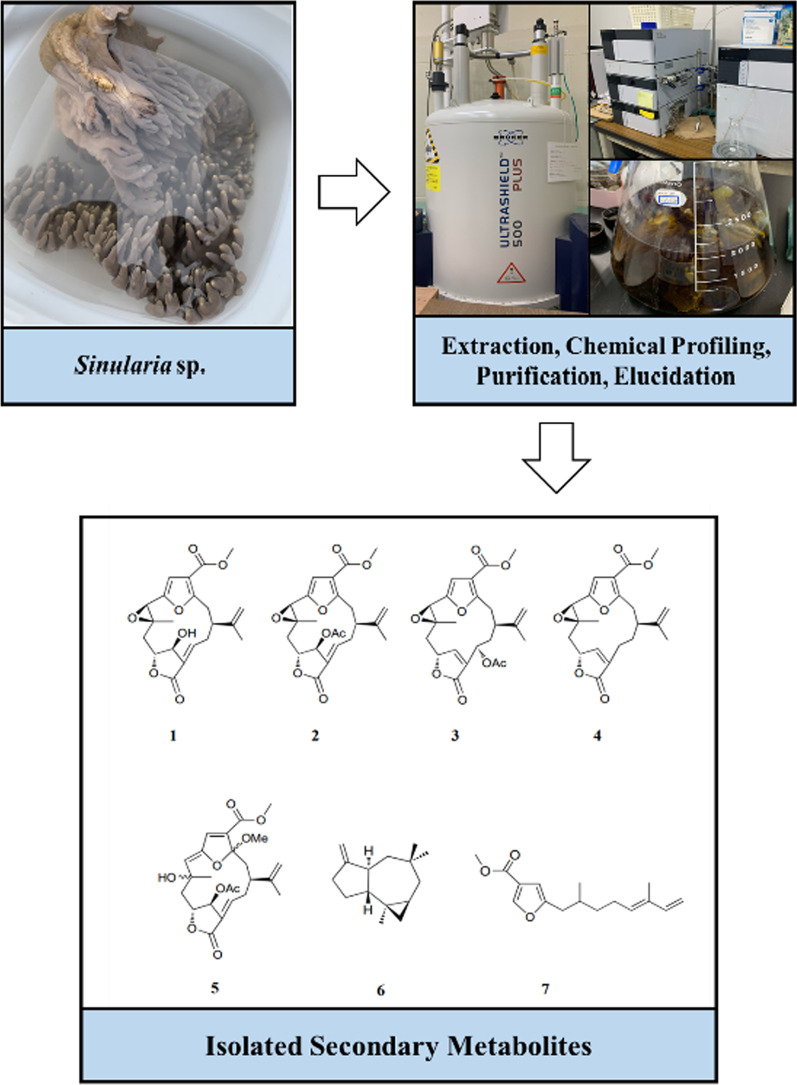

**Supplementary Information:**

The online version contains supplementary material available at 10.1007/s13659-022-00330-7.

## Introduction

The soft coral genus *Sinularia* (phylum Cnidaria, class Anthozoa, subclass Octocorallia, order Alcyonacea, family Alcyoniidae) is one of the most widely distributed soft coral genera in the tropics and subtropics, including Okinawa, Japan, inhabiting coral reefs or rocks in shallow waters [1, 2]. Over the past 50 years, bioactive compounds, particularly various types of secondary metabolites such as sesquiterpenoids and diterpenoids, have been isolated from several species of the genus *Sinularia*, which makes them attractive targets for extensive chemical and biomedical research. In addition, more than 500 secondary metabolites of different biological origins have been identified in approximately 50 *Sinularia* species [3, 4]. A significant number of these metabolites exhibit potent biological properties, including cytotoxic, antibacterial, antifungal, anti-inflammatory, and immunosuppressive activities [5–9].

**Fig. 1:**
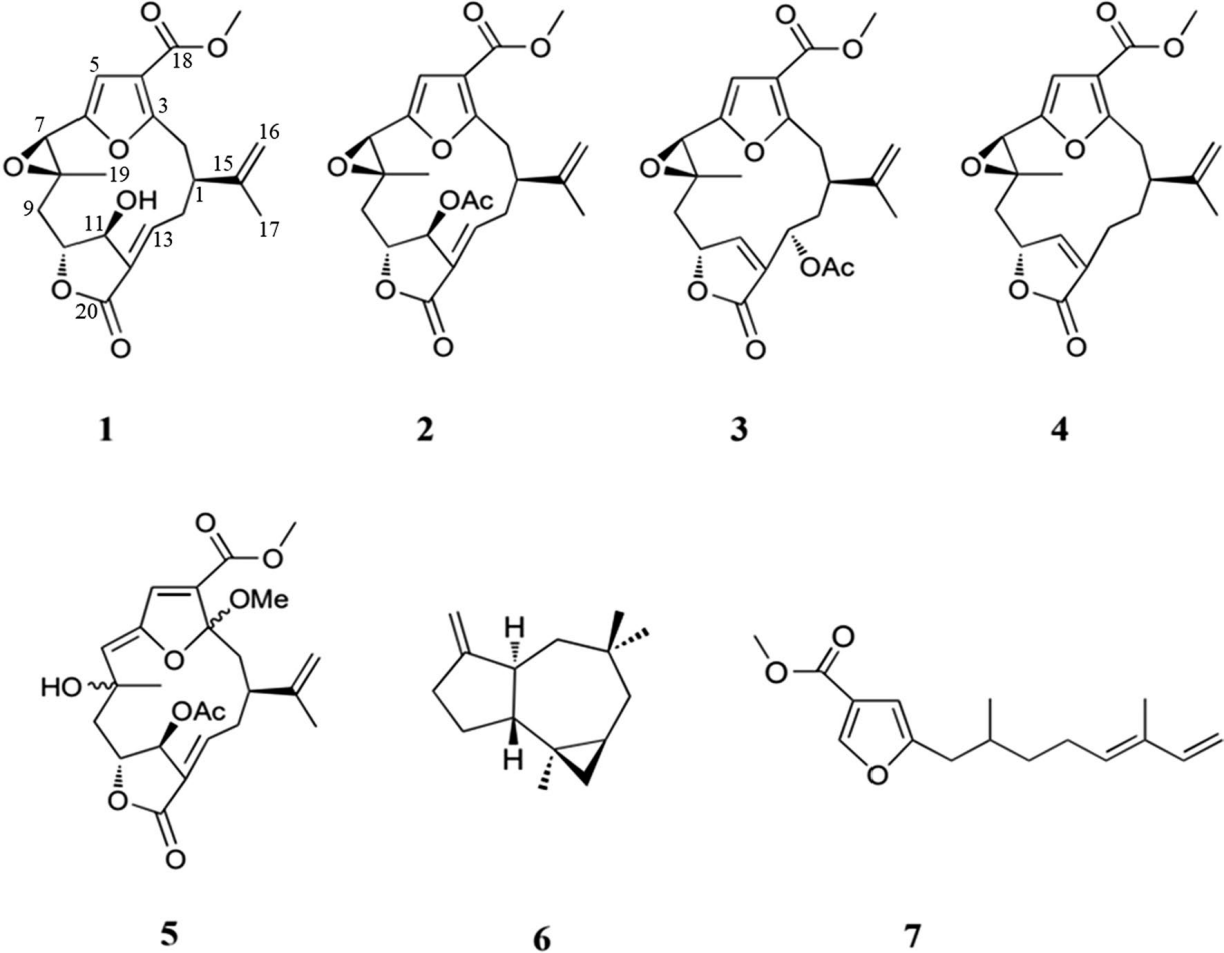
Chemical structures of compounds **1–7**

This genus of *Sinularia* has also been studied for its chemical composition and biological activity in Okinawa, and various novel bioactive compounds have been isolated [10–12]. As part of our continuous research on bioactive compounds, a new compound, 11-hydroxy-Δ^12(13)^-pukalide (**1**), along with six known secondary metabolites, 11-acetoxy-Δ^12(13)^-pukalide (**2**), 13α-acetoxypukalide (**3**), pukalide (**4**), 3α-methoxyfuranocembranoid (**5**), Δ^9(15)^-africanene (**6**), and methyl (5′*E*)-5-(2′,6′-dimethylocta-5′,7′-dienyl)furan-3-carboxylate (**7**) (Fig. 1), were isolated from the Okinawan soft coral *Sinularia* sp. In addition, we examined the antibacterial activities of *Ralstonia solanacearum* MAFF730131, along with toxic activities using the brine shrimp lethality test of the isolated compounds **1**–**7**.

## Results and discussion

Compound **1** was isolated as a yellow oil with [*α*]_D_^27^‒215 (*c* 0.1, CHCl_3_). Its molecular formula was established as C_21_H_24_O_7_ based on HRESIMS, the positive ion at *m/z* 389.1595 [M + H]^+^ (calcd 389.1600), indicating 10 degrees of unsaturation. The IR spectrum revealed the presence of hydroxy (3471 cm^−1^) and carbonyl functionalities (1715 cm^−1^). The ^1^H and ^13^C NMR spectra of **1** (Table 1) indicated the presence of 21 carbon signals, where their multiplicities were confirmed by DEPT and HSQC measurements as three methyls (including one methoxy), three *sp*^3^ methylenes, four *sp*^3^ methines (including three oxymethines), one *sp*^2^ methylene, two *sp*^2^ methines, and eight quaternary carbons. Comparison with the data of similar functionality in previous reports further supported that compound **1** is typical of a furanocembranoid [13–15]. In addition, the careful examination of ^1^H and ^13^C NMR spectra (Table 1) revealed that the structures of **1** and **2** were identical except for the replacement of an acetoxy group at C-11 in **2** by a hydroxy group in **1**.

**Table 1:** ^13^C NMR (125 MHz) and ^1^H NMR (500 MHz) spectroscopic data for compound **1** (δ in ppm and *J* in Hz) in CDCl_3_

No	*δ*_C_	*δ*_H_ (Mult. *J*)
1	41.0	2.87–2.90 (m)
2	30.7	2.97 (dd, 15.5, 5.2) 3.56 (dd, 15.5, 5.2)
3	160.5	–
4	114.3	–
5	108.1	6.35 (d, 1.0)
6	147.9	–
7	54.4	3.89 (d, 1.0)
8	56.9	–
9	40.8	1.85 (dd, 15.2, 3.6) 2.42 (dd, 15.2, 3.6)
10	83.6	4.59 (t, 3.6)
11	72.3	4.64 (s)
12	128.4	–
13	148.8	6.52 (dd, 11.4, 2.8)
14	30.5	2.60 (ddd, 17.6, 8.5, 2.8) 3.95 (ddd, 17.6, 11.4, 1.7)
15	145.2	–
16	112.5	4.56 (s) 4.82 (s)
17	23.4	1.68 (s)
18	164.0	–
19	21.5	1.10 (s)
20	169.0	-
OMe	51.4	3.73 (s)

**Fig. 2:**
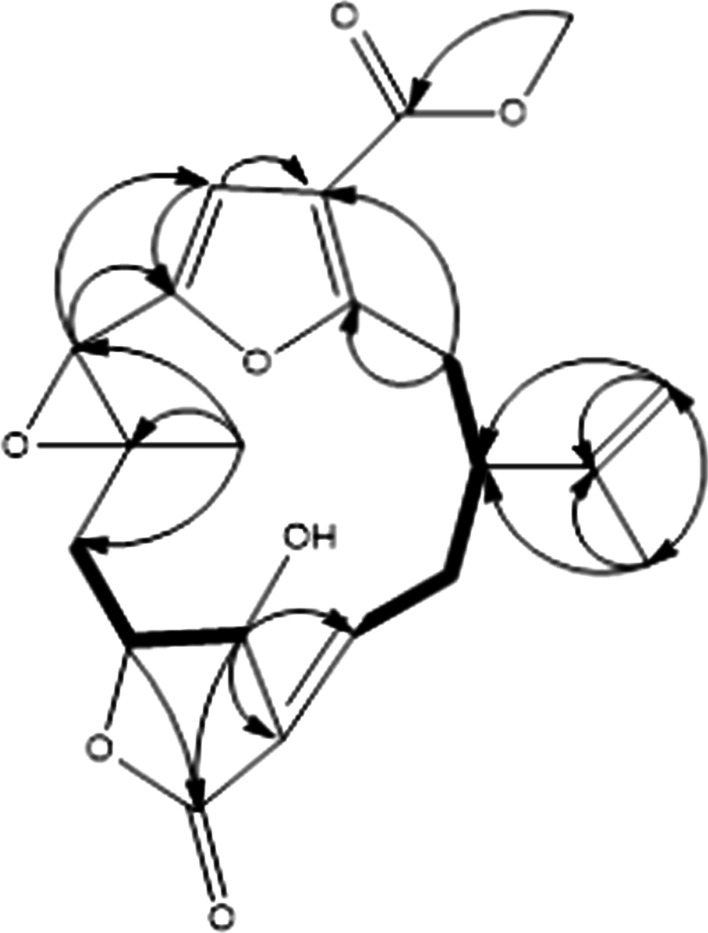
^1^H–^1^H COSY (bold lines) and selective HMBC (arrows) of compound **1**

The ^1^H–^1^H COSY (Fig. 2) experiment indicated two sequences of correlated protons, H-C(13)/H_2_-C(14)/H-C(1)/H_2_-C(2), and H_2_-C(9)/H-C(10)/H-C(11). The skeleton of compound **1** was deduced a furanocembranoid diterpene with a γ-lactone moiety in the HMBC experiment (Fig. 2) of H_2_-2 to C-3 and C-4; H-5 to C-4 and C-6; H-7 to C-5 and C-6; H-10 to C-20; H-11 to C-12, C-13, and C-20; H_3_-19 to C-7, C-8, and C-9. In addition, the HMBC spectra of H_2_-16 to C-1, C-15, and C-17; H_3_-17 to C-1, C-15, and C-16 confirmed the position of the isopropyl group.

The relative stereochemistry of **1** was deduced from the NOESY correlation and comparison of its NMR spectrum, coupling constant, and NOE correlation with those of known analogs. The coupling constant (*J*_10,11_ ≈ 0 Hz) suggested that the hydrogens were disposed to each other with a dihedral angle of 90° between H-10 and H-11. This confirmed the *trans* orientation of H-10 and H-11 [16]. The NOE correlations for H-11 and H-13 indicated that the double bond between C-12 and C-13 was in the (*Z*)-configuration. Furthermore, the steric structure of compound **1** was determined because the coupling constants of compounds **1** and **2** were identical. In addition, the ^1^H and ^13^C NMR spectra of acetylated compound **1** were consistent with those of compound **2**. Thus, the relative stereochemistry of **1** was assigned to be the same as that of **2**. To determine the absolute configuration of natural product **1**, the modified Mosher’s analysis of **1** is ongoing in our laboratory.

The structures of known compounds were identified as 11-acetoxy-Δ^12(13)^-pukalide (**2**) [16], 13α-acetoxypukalide (**3**) [16], pukalide (**4**) [17], 3α-methoxyfuranocembranoid (**5**) [18], Δ^9(15)^-africanene (**6**) [19], and methyl (5′*E*)-5-(2′,6′-dimethylocta-5′,7′-dienyl)furan-3-carboxylate (**7**) [20], by comparing their spectroscopic data with those reported in the literature.

The antibacterial activities of compounds **1**–**7** were evaluated against the phytopathogens *R. solanacearum* MAFF730131. Unfortunately, none of the compounds exhibited any antibacterial activity. In addition, the toxicities of compounds **1**–**7** were tested against brine shrimp. Consequently, compounds **1** and **7** were toxic against *Artemia salina* with LC_50_ 47.5 and 24.6 µg/mL, respectively, whereas the other compounds exhibited negligible effects with LC_50_ > 100 µg/mL.

## Experimental

### General experimental procedures

Optical rotation was measured using a P-1010 polarimeter (Jasco) in chloroform at 27 °C. IR spectra were recorded on a FT/IR-6100 spectrometer (Jasco). NMR spectra were recorded on a 500 MHz NMR AVANCE III (Bruker) using deuterated chloroform (CDCl_3_) and deuterated benzene (C_6_D_6_). MS spectra were obtained using a SYNAPT HDMS system (Waters). Preparative TLC was performed using silica gel plates (Merck Kieselgel 60 F_254_). Silica gel (Kanto Chemical, Silica gel 60 N, spherical, neutral, 100–210 µm) was used for column chromatography. Semi-preparative HPLC was performed on a Shimadzu HPLC system with a Cosmosil πNAP (10 × 250 mm) column.

### Animal materials

Specimens of *Sinularia* sp. were collected from the coast of Minato-Machi (26°13′55"N, 127°40′17"E), Naha, Okinawa, Japan, on November 13, 2019. The voucher specimen was deposited at the Faculty of Agriculture, University of the Ryukyus.

### Extraction and isolation

The soft coral *Sinularia* sp. specimens (1.25 kg, wet wt) were sliced and extracted with 100% methanol (MeOH) for one week at 25 °C. The resulting crude extract was concentrated *in vacuo* and partitioned between ethyl acetate (EtOAc)/distilled water (H_2_O). The EtOAc fraction (7.14 g) was further partitioned with *n*-hexane/90% MeOH to obtain *n*-hexane (3.15 g) and 90% MeOH (3.42 g) fractions. The *n*-hexane and 90% MeOH fractions were subjected to silica gel column chromatography elution with a gradient of *n*-hexane/EtOAc (9:1, 8:2, 7:3, 5:5, and 0:10) to yield five fractions 1–5. The *n*-hexane fraction 1 (28.4 mg) was further separated by preparative TLC with *n*-hexane to yield **6** (22.6 mg). The MeOH fraction 2 (25.6 mg) yielded **7** (10.5 mg) after purification by preparative TLC using *n*-hexane/EtOAc (1:1) and toluene. MeOH fraction 4 (739.1 mg) was further separated by preparative TLC with *n*-hexane/EtOAc (1:1) to afford **2** (15.7 mg). In addition, MeOH fraction 5 (571.3 mg) was subjected to preparative TLC with *n*-hexane/EtOAc (1:1) and toluene/EtOAc (1:1) to yield **1** (18.8 mg) and **3** (19.1 mg), which were further purified by preparative HPLC to yield **4** (1.9 mg) and **5** (2.4 mg). The isolation was performed using a πNAP column measured at an UV wavelength of 210 nm under 70% and 80% MeOH.

#### 11-Hydroxy-Δ^12(13)^-pukalide (1)

Yellow oil; [*α*]_D_^27^ ‒215 (*c* 0.1, CHCl_3_); IR (liquid film) ν_max_ 3477, 2926, 1746, 1717, 1442, 1385, 1229, 1077, 757 cm^−1^; ^1^H NMR (CDCl_3_, 500 MHz) δ_H_: 6.52 (1H, dd, *J* = 11.4, 2.8 Hz, H-13), 6.35 (1H, d, *J* = 1.0 Hz, H-5), 4.82 (2H, s, H-16), 4.64 (1H, s, H-11), 4.59 (1H, t, *J* = 3.6 Hz, H-10), 4.56 (2H, s, H-16), 3.95 (2H, ddd, *J* = 17.6, 11.4, 1.7 Hz, H-14), 3.89 (1H, d, *J* = 1.0 Hz, H-7), 3.73 (3H, s, 18-OMe), 3.56 (2H, dd, *J* = 15.5, 5.2 Hz, H-2), 2.97 (2H, dd,* J* = 15.5, 5.2 Hz, H-2), 2.87–2.90 (1H, m, H-1), 2.60 (2H, ddd, *J* = 17.6, 8.5, 2.8 Hz, H-14), 2.42 (2H, dd, *J* = 15.2, 3.6 Hz, H-9), 1.85 (2H, dd, *J* = 15.2, 3.6 Hz, H-9), 1.68 (3H, s, H-17), 1.10 (3H, s, H-19); ^13^C NMR (CDCl_3_, 125 MHz) δ_C_: 169.0 (C, C-20), 164.0 (C, C-18), 160.5 (C, C-3), 148.8 (CH, C-13), 147.9 (C, C-6), 145.2 (C, C-15), 128.4 (C, C-12), 114.3 (C, C-4), 112.5 (CH_2_, C-16), 108.1 (CH, C-5), 83.6 (CH, C-10), 72.3 (CH, C-11), 56.9 (C, C-8), 54.4 (CH, C-7), 51.4 (CH_3_, 18-OMe), 41.0 (CH, C-1), 40.8 (CH_2_, C-9), 30.7 (CH_2_, C-2), 30.5 (CH_2_, C-14), 23.4 (CH_3_, C-17), 21.5 (CH_3_, C-19); HRESIMS *m/z* 389.1595 [M + H]^+^ (calcd for C_21_H_25_O_7_, 389.1600).

### Acetylation of 11-hydroxy-Δ^12(13)^-pukalide (1)

Compound **1** (1 mg) was acetylated with acetic anhydride (72 µL) and 4-dimethylaminopyridine (4 mg) in dichloromethane (CH_2_Cl_2_). The mixture was stirred at 0 °C overnight, and thereafter partitioned with CH_2_Cl_2_/H_2_O to afford **2** (1 mg), which exhibited HRESIMS as the positive ion at *m/z* 431.1706 [M + H]^+^ (calcd for C_23_H_27_O_8_, 431.1706).

### Bioassay

#### Antibacterial assay

*Ralstonia solanacearum* was streaked onto casamino acids peptone glucose (CPG) agar (peptone 10.0 g, casamino acids 1.0 g, glucose 5.0 g, agar 17.0 g, and deionized water 1 L) from − 80 °C glycerol stocks and grown at 30 °C for 48 h to obtain a single colony. It was transferred into CPG broth and grown at 28 °C with shaking at 225 rpm for 48 h to the exponential growth phase (optical density at 660 nm [OD_660_] = 0.1) [21]. Its bacterial solution was added to Top agar (peptone 3.0 g, casamino acids 0.3 g, glucose 1.7 g, agar 5.0 g, and deionized water 1 L) and poured onto CPG agar medium and allowed to solidify. The isolated compounds dissolved in MeOH (1 mg/mL) were impregnated on sterile filter paper discs (6 mm disc diameter) and thereafter applied aseptically to the surface of the agar plates. Chloramphenicol was used as the positive control. The plates were subsequently incubated at 30 °C for 24 h. Then, the diameters of the inhibition zone including the 6 mm disc diameter, were measured. Experiments were conducted in triplicate, and the results were presented as mean values [22].

#### Brine shrimp toxicity assay

The eggs of brine shrimp (*Artemia salina*) were hatched in artificial seawater (prepared by dissolving instant sea salt (13.5 g) in 450 mL of distilled water) at room temperature. After 48 h, the phototropic nauplii were collected, and 10 shrimp were transferred to each sample vial using a pipette. The isolated compounds were bioassayed in 1.5 mL tubes containing 1 mL of 10 brine shrimps at a final concentration of 100 µg/mL. After 24 h, the number of surviving shrimp was counted, and only those compounds that exceeded 50% lethality were bioassayed again at final concentrations of 10, 20, and 50 µg/mL. Dimethyl sulfoxide was used as a negative control. The mortality rate at each concentration was calculated to determine LC_50_ values. Experiments were conducted in triplicate, and the results were presented as mean values [23].

## Supplementary Information

**Additional file 1: Figure S1.** ^1^H NMR spectrum of 11-hydroxy-Δ^12(13)^-pukalide (**1**) in CDCl_3_ (500 MHz). **Figure S2.** ^13^C NMR spectrum of 11-hydroxy-Δ^12(13)^-pukalide (**1**) in CDCl_3_ (125 MHz). **Figure S3.** DEPT135 spectrum of 11-hydroxy-Δ^12(13)^-pukalide (**1**) in CDCl_3_. **Figure S4.** ^1^H–^1^H COSY spectrum of 11-hydroxy-Δ^12(13)^-pukalide (**1**) in CDCl_3_. **Figure S5. **HSQC spectrum of 11-hydroxy-Δ^12(13)^-pukalide (**1**) in CDCl_3_. **Figure S6.** HMBC spectrum of 11-hydroxy-Δ^12(13)^-pukalide (**1**) in CDCl_3_. **Figure S7.** HRESIMS of 11-hydroxy-Δ^12(13)^-pukalide (**1**). **Figure S8.** IR spectrum of 11-hydroxy-Δ^12(13)^-pukalide (**1**).
