# Supplementary material for: Furanocembranoid from the Okinawan soft coral Sinularia sp
Source: Nat Prod Bioprospect. 2022 Mar 2;12(1):7. doi: 10.1007/s13659-022-00330-7 (PMC8888784; doi:10.1007/s13659-022-00330-7)
Supplement: Supplementary file 1 — Additional file 1: Figure S1. 1H NMR spectrum of 11-hydroxy-Δ12(13)-pukalide (1) in CDCl3 (500 MHz). Figure S2. 13C NMR spectrum of 11-hydroxy-Δ12(13)-pukalide (1) in CDCl3 (125 MHz). Figure S3. DEPT135 spectrum of 11-hydroxy-Δ12(13)-pukalide (1) in CDCl3. Figure S4. 1H–1H COSY spectrum of 11-hydroxy-Δ12(13)-pukalide (1) in CDCl3. Figure S5. HSQC spectrum of 11-hydroxy-Δ12(13)-pukalide (1) in CDCl3. Figure S6. HMBC spectrum of 11-hydroxy-Δ12(13)-pukalide (1) in CDCl3. Figure S7. HRESIMS of 11-hydroxy-Δ12(13)-pukalide (1). Figure S8. IR spectrum of 11-hydroxy-Δ12(13)-pukalide (1). [file 13659_2022_330_MOESM1_ESM.docx]

**Supplementary material**

Furanocembranoid from the Okinawan soft coral *Sinularia* sp.

Misaki Nagasaka ^a^, Kazuki Tani ^a^, Keisuke Nishikawa ^b^, Riri Kinjo ^a^, and Takahiro Ishii ^a*^

^a^ Department of Biosciences and Biotechnology, Faculty of Agriculture, University of the Ryukyus,

Senbaru 1, Nishihara, Okinawa 903-0213, Japan. E-Mail: ishiit@agr.u-ryukyu.ac.jp (T.I.)

^b^ Department of Chemistry, Graduate School of Science, Osaka City University, Osaka 558-8585, Japan

**List of Figures**

Figure S1. ^1^H NMR spectrum of 11-hydroxy-Δ^12(13)^-pukalide (**1**) in CDCl_3_ (500 MHz).

Figure S2. ^13^C NMR spectrum of 11-hydroxy-Δ^12(13)^-pukalide (**1**) in CDCl_3_ (125 MHz).

Figure S3. DEPT135 spectrum of 11-hydroxy-Δ^12(13)^-pukalide (**1**) in CDCl_3_.

Figure S4. ^1^H–^1^H COSY spectrum of 11-hydroxy-Δ^12(13)^-pukalide (**1**) in CDCl_3_.

Figure S5. HSQC spectrum of 11-hydroxy-Δ^12(13)^-pukalide (**1**) in CDCl_3_.

Figure S6. HMBC spectrum of 11-hydroxy-Δ^12(13)^-pukalide (**1**) in CDCl_3_.

Figure S7. HRESIMS of 11-hydroxy-Δ^12(13)^-pukalide (**1**).

Figure S8. IR spectrum of 11-hydroxy-Δ^12(13)^-pukalide (**1**).

Figure S1. ^1^H NMR spectrum of 11-hydroxy-Δ^12(13)^-pukalide (**1**) in CDCl_3_ (500 MHz).

**
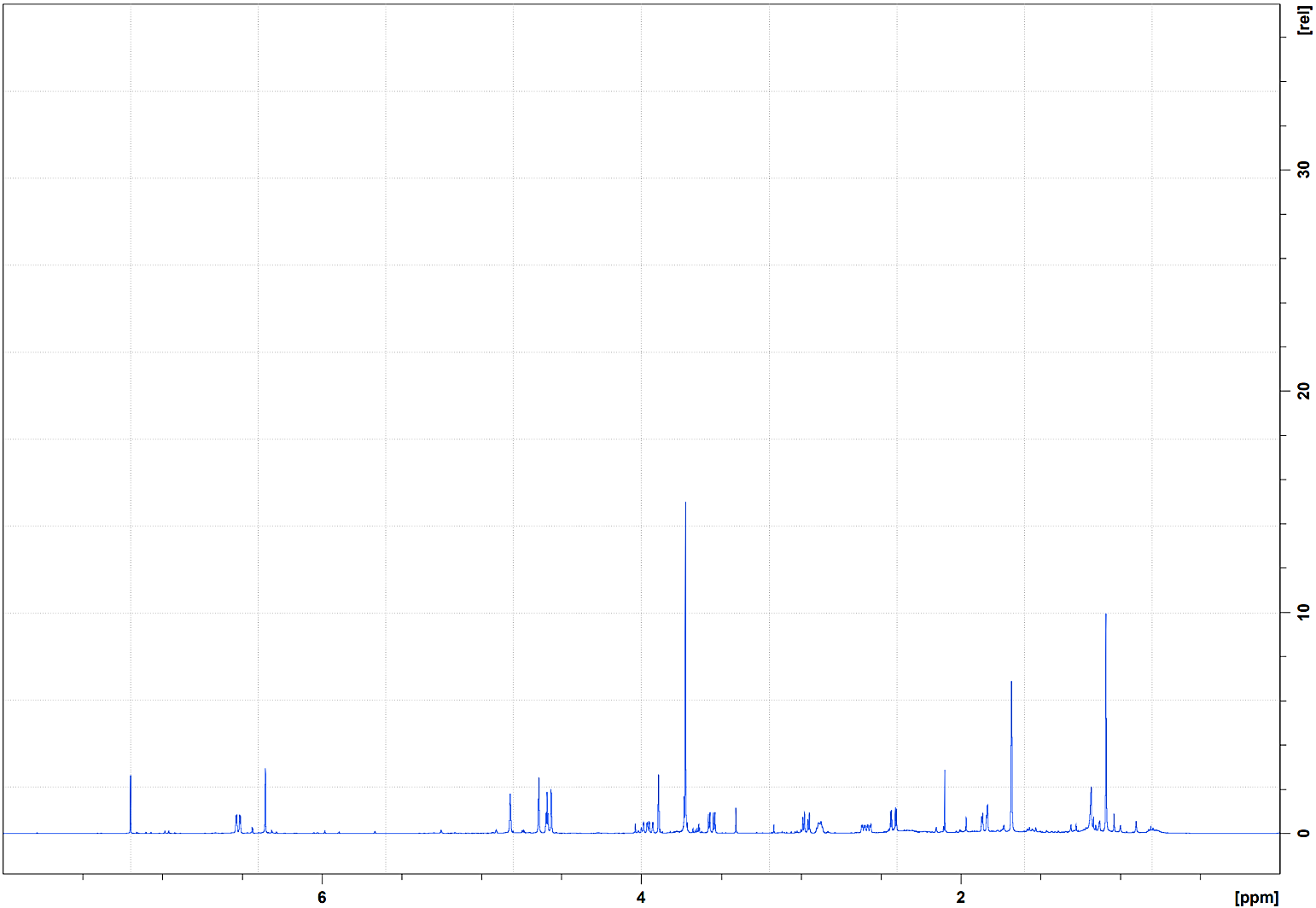
**Figure S2. ^13^C NMR spectrum of 11-hydroxy-Δ^12(13)^-pukalide (**1**) in CDCl_3_ (125 MHz).

**
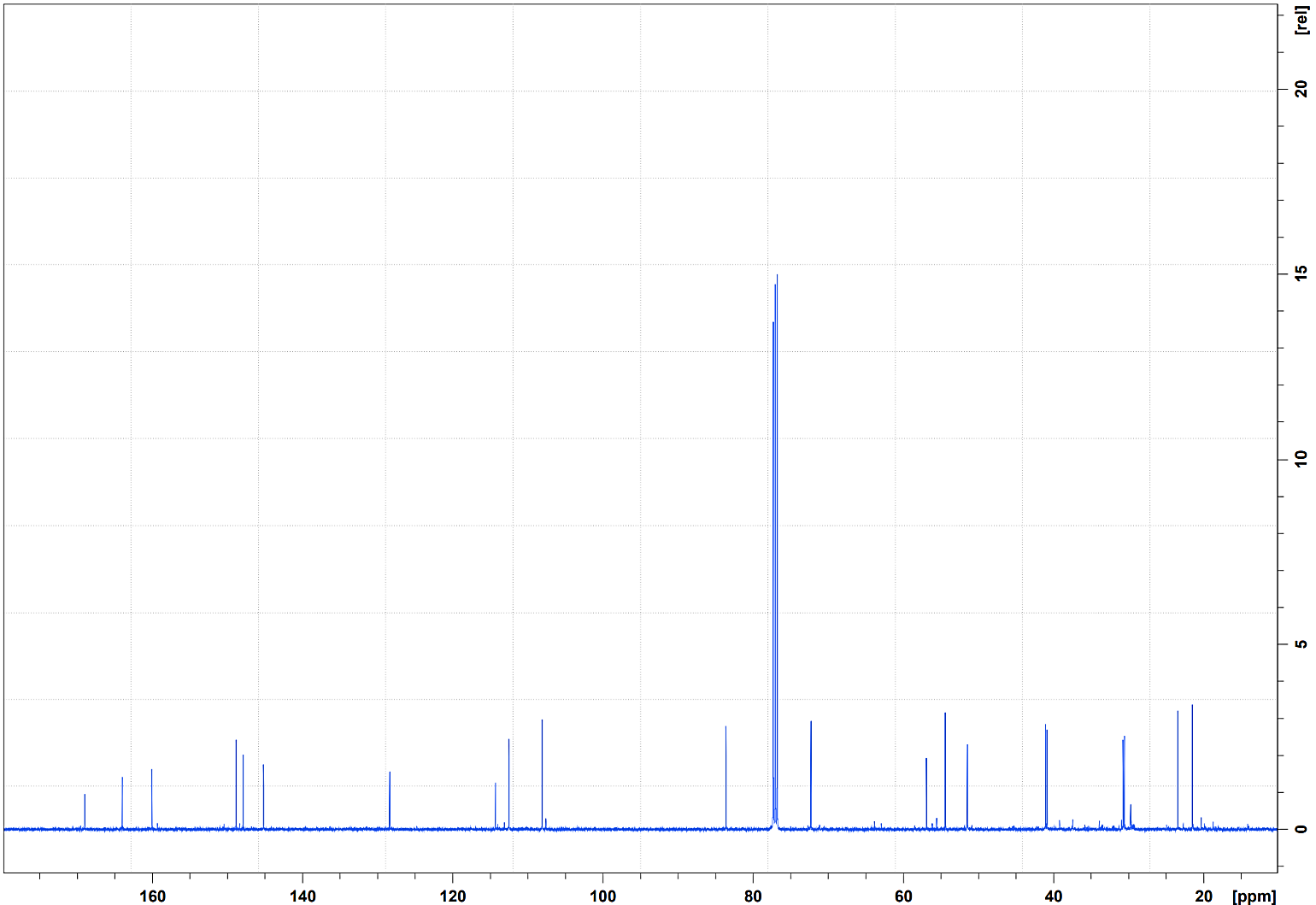
**

Figure S3. DEPT135 spectrum of 11-hydroxy-Δ^12(13)^-pukalide (**1**) in CDCl_3_.

**
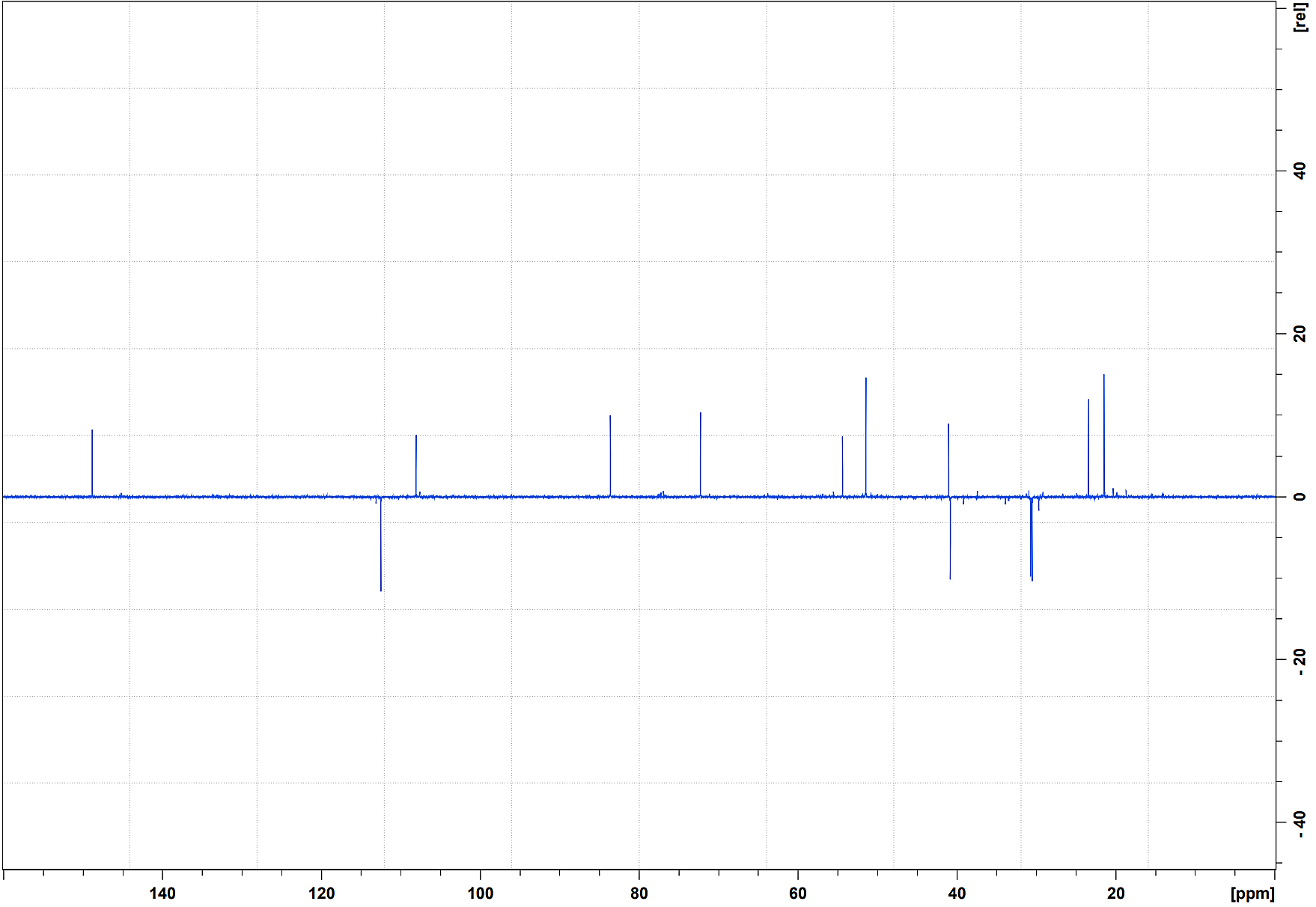
**

Figure S4. ^1^H–^1^H COSY spectrum of 11-hydroxy-Δ^12(13)^-pukalide (**1**) in CDCl_3_.

**
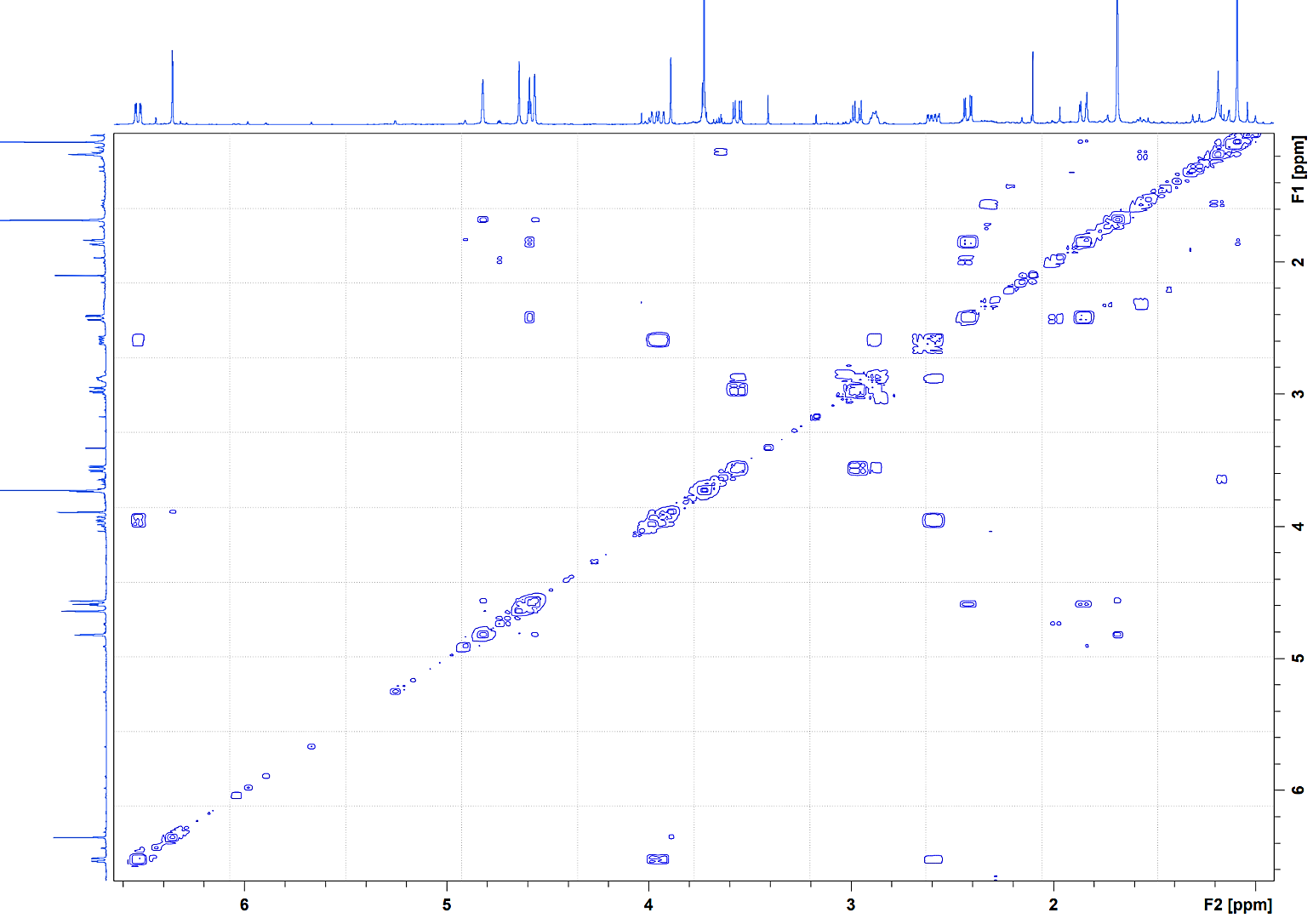
**

Figure S5. HSQC spectrum of 11-hydroxy-Δ^12(13)^-pukalide (**1**) in CDCl_3_.

**
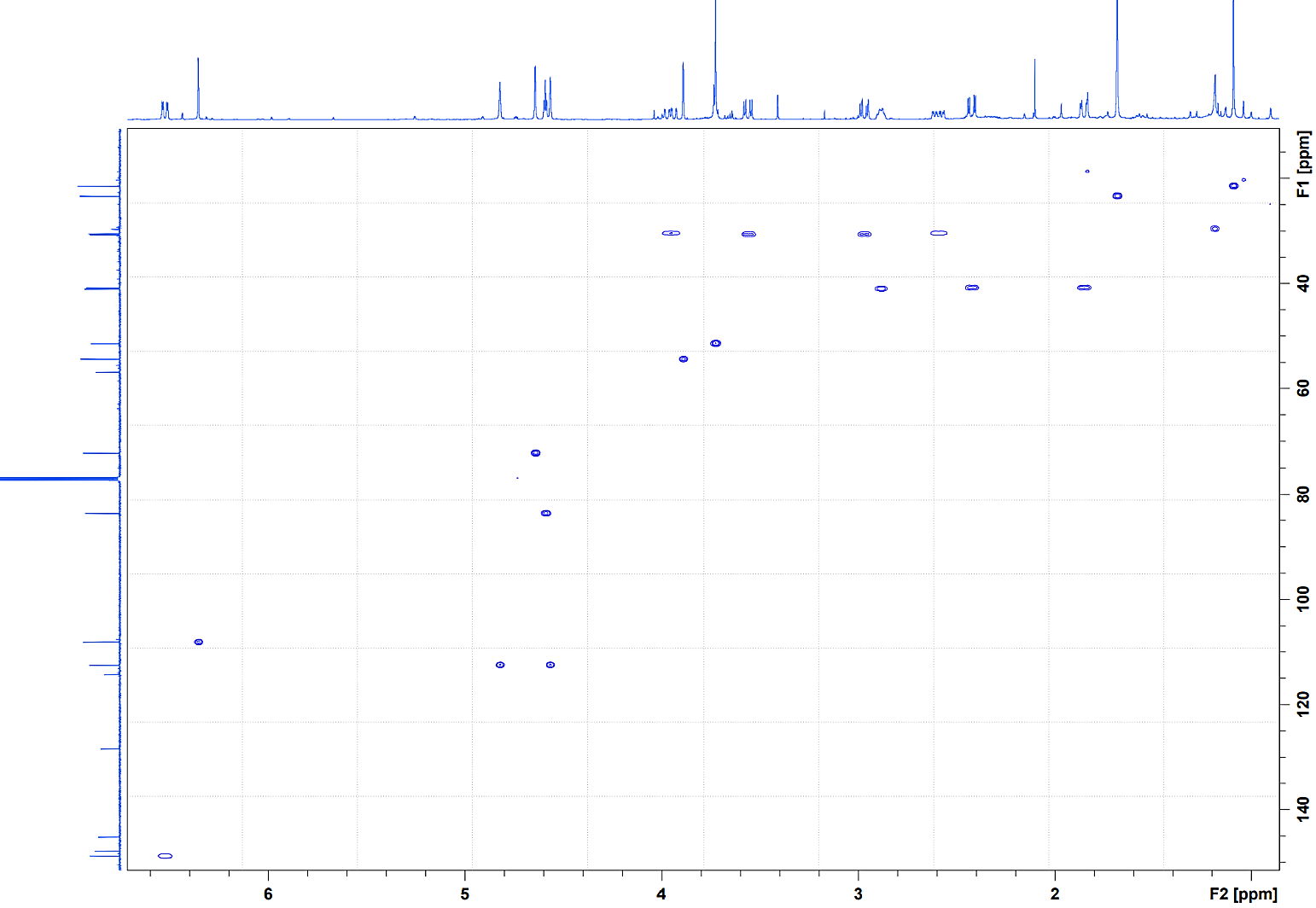
**

Figure S6. HMBC spectrum of 11-hydroxy-Δ^12(13)^-pukalide (**1**) in CDCl_3_.

**
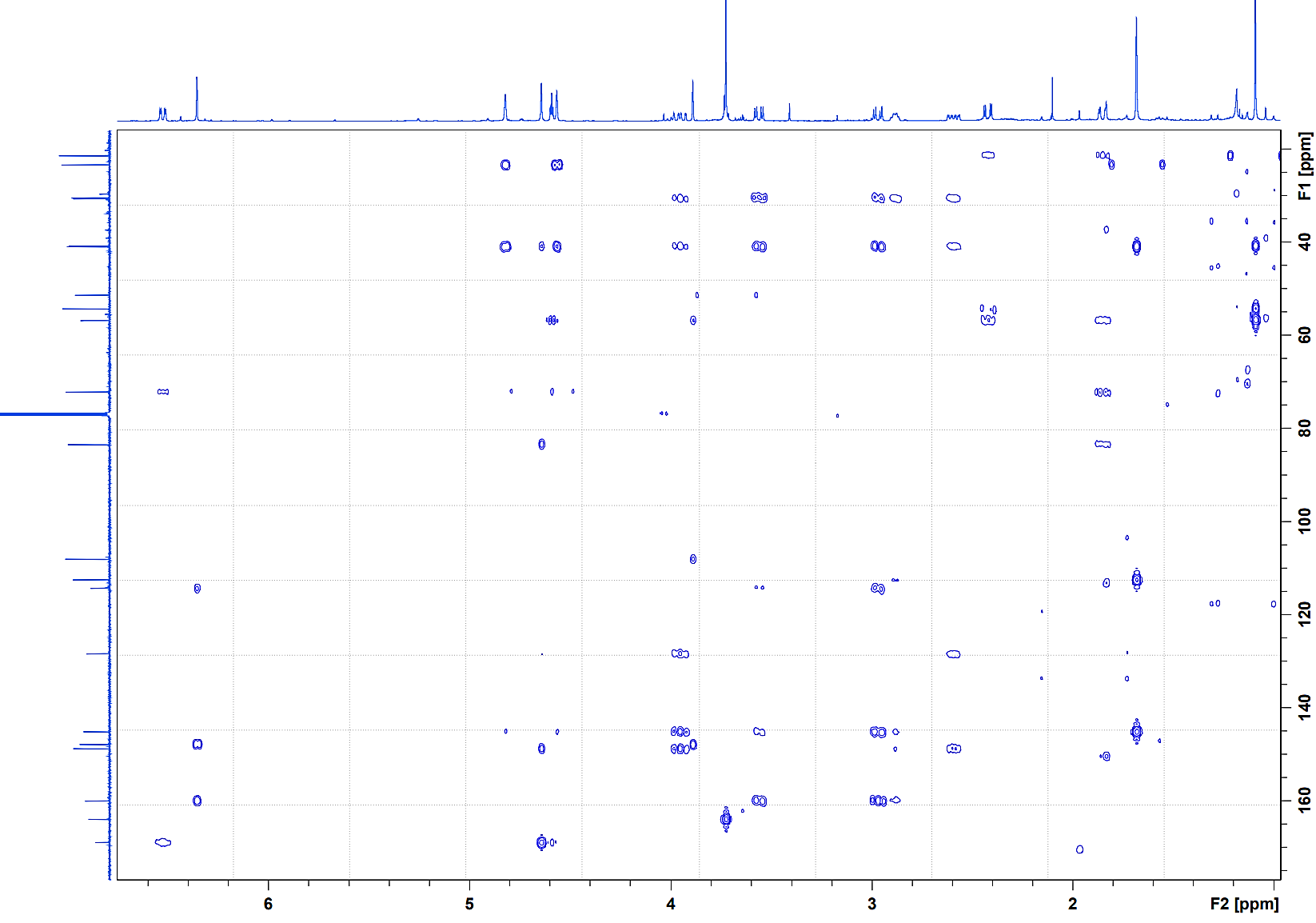
**

Figure S7. HRESIMS of 11-hydroxy-Δ^12(13)^-pukalide (**1**).

**
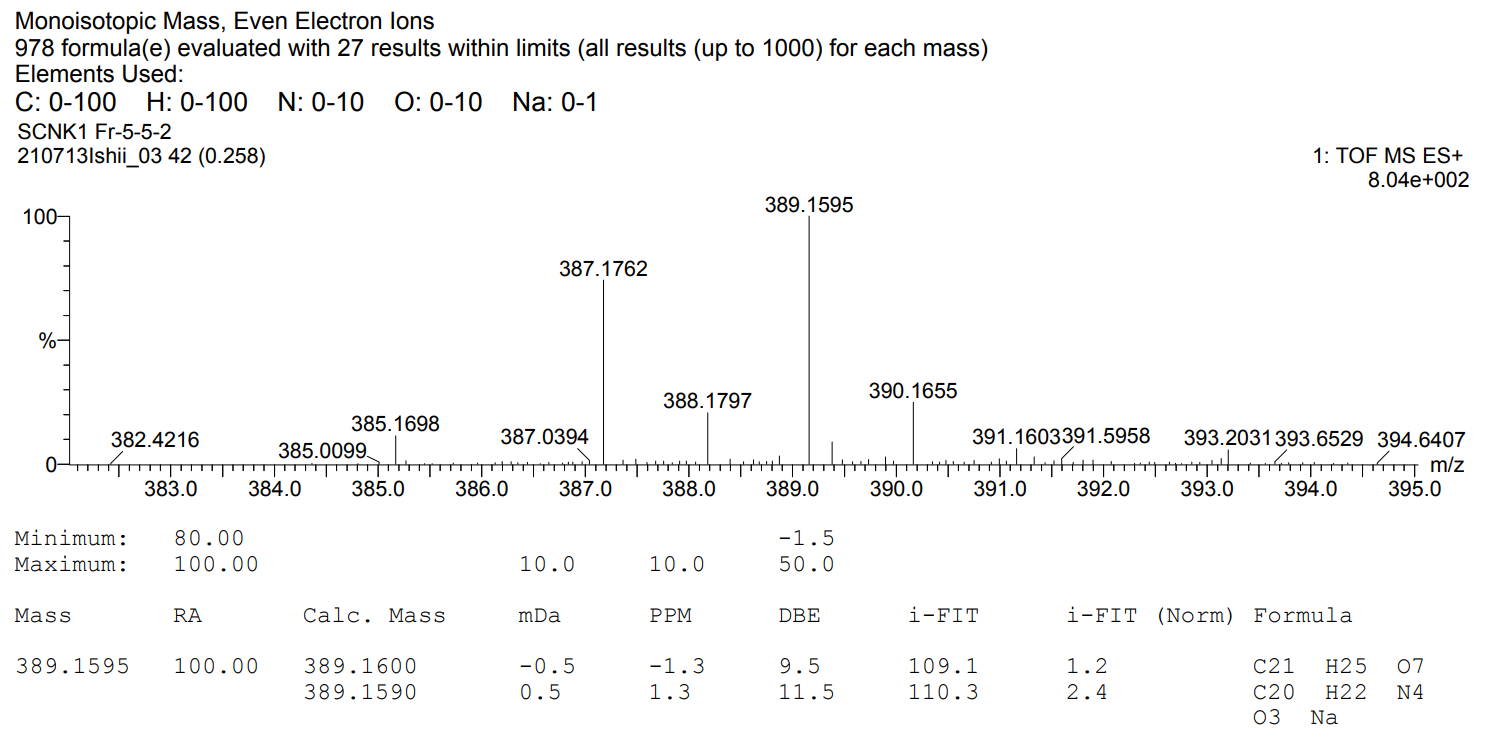
**

Figure S8. IR spectrum of 11-hydroxy-Δ^12(13)^-pukalide (**1**).

**
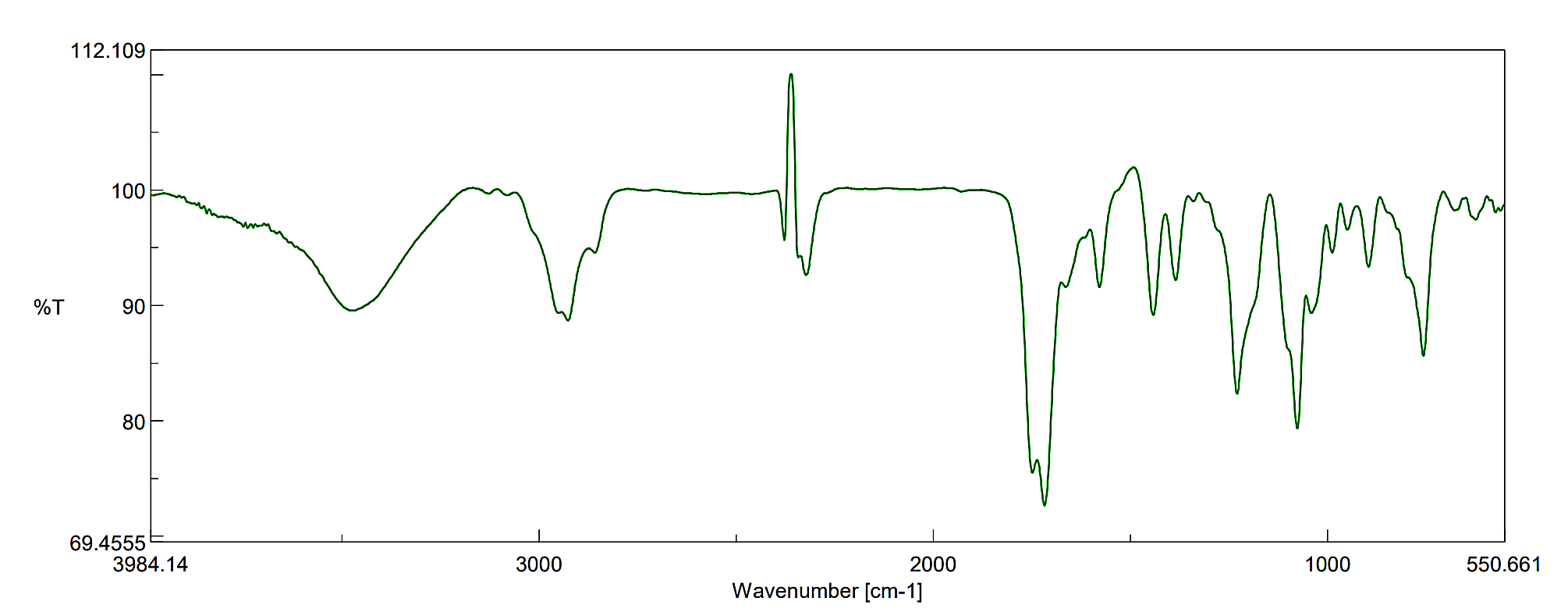
**
